# Pheochromocytoma presenting with hyperosmolar hyperglycemic state and transient stroke-like deficits

**DOI:** 10.1210/jcemcr/luag266

**Published:** 2026-09-16

**Authors:** Alaa Almallouhi, Mustafa Sadek, Edward Ruby

**Affiliations:** Internal Medicine Residency Program, Naples Comprehensive Health, Naples, FL 34102, USA; Internal Medicine Residency Program, Naples Comprehensive Health, Naples, FL 34102, USA; Internal Medicine Residency Program, Naples Comprehensive Health, Naples, FL 34102, USA

**Keywords:** pheochromocytoma, hyperosmolar hyperglycemic state, stroke mimic, catecholamine excess, adrenalectomy

## Abstract

Catecholamine excess can disrupt glucose metabolism, but presentation with hyperosmolar hyperglycemic state and transient focal neurologic deficits is uncommon. A 37-year-old man with obesity, hypertension, and prediabetes presented with slurred speech and right arm weakness. Serum glucose was 1252 mg/dL (International System of Units (SI): 69.5 mmol/L) (reference range, 70-99 mg/dL [SI: 3.9-5.5 mmol/L]), serum osmolality was 325 mOsm/kg (reference range, 275-295 mOsm/kg), and hemoglobin A1c was 16.1% (SI: 152 mmol/mol) (reference range, 4.0-5.6% [SI: 20-38 mmol/mol]). Computed tomography angiography and perfusion imaging showed no cerebral ischemia, and the deficits resolved with correction of hyperosmolarity. Because of severe hypertension and abrupt deterioration in glycemic control, plasma metanephrines were measured and found to be markedly elevated, prompting imaging that identified an 8.4-cm right adrenal mass. After α-adrenergic blockade and glycemic optimization, robotic adrenalectomy was performed. Pathology confirmed a 9-cm pheochromocytoma with classic zellballen architecture and a Ki-67 index of approximately 1%. Within 3 weeks, hypertension and insulin-requiring diabetes resolved, allowing discontinuation of all antihypertensive and glucose-lowering medications. This case highlights pheochromocytoma as a reversible cause of severe dysglycemia and a probable metabolic stroke mimic.

## Introduction

Pheochromocytomas are catecholamine-producing adrenal medullary neuroendocrine neoplasms with highly variable clinical manifestations. Catecholamine excess can impair glucose homeostasis by suppressing insulin secretion, increasing hepatic glucose production, and worsening peripheral insulin resistance; glycemic disorders affect more than one-third of patients and often improve after tumor resection [1-3]. Hyperosmolar hyperglycemic state (HHS) is an uncommon presentation [4], and HHS-associated focal neurologic deficits may resemble acute ischemic stroke [5]. We report a large functional pheochromocytoma presenting with HHS and transient stroke-like deficits, followed by rapid resolution of hypertension and insulin-requiring diabetes after adrenalectomy.

## Case presentation

A 37-year-old Black man with class III obesity (body mass index, 51.8 kg/m2), hypertension, hyperlipidemia, obstructive sleep apnea, nonobstructive coronary artery disease, and prediabetes presented after 4 days of lethargy, weakness, and lightheadedness followed by acute slurred speech and right arm weakness. On retrospective review, the patient did not report a prominent or recurrent constellation of classic pheochromocytoma symptoms before presentation that had prompted evaluation for a catecholamine-secreting tumor. Hemoglobin A1c 6 months earlier was 6.1% (International System of Units (SI): 43 mmol/mol) (reference range, 4.0-5.6% [SI: 20-38 mmol/mol]). On arrival, blood pressure was 199/135 mmHg and heart rate was 117 beats/min. A stroke alert was activated; the National Institutes of Health Stroke Scale score was 0 at formal assessment.

## Diagnostic assessment

Serum glucose was 1252 mg/dL (SI: 69.5 mmol/L) (reference range, 70-99 mg/dL [SI: 3.9-5.5 mmol/L]), measured osmolality was 325 mOsm/kg (reference range, 275-295 mOsm/kg), and hemoglobin A1c was 16.1% (SI: 152 mmol/mol). Bicarbonate and the anion gap were not consistent with ketoacidosis. Creatinine was 2.1 mg/dL (SI: 186 μmol/L) (reference range, 0.7-1.3 mg/dL [SI: 62-115 μmol/L]) and peaked at 2.6 mg/dL (SI: 230 μmol/L). These findings supported HHS with acute kidney injury.

Noncontrast head computed tomography (CT) showed no hemorrhage or acute infarction. Computed tomography angiography showed no large-vessel occlusion or high-grade stenosis, and CT perfusion showed neither ischemic core nor penumbra. The deficits resolved as hyperosmolarity corrected. Brain magnetic resonance imaging was deferred, and the episode was considered a probable metabolic stroke mimic.

**Figure 1:**
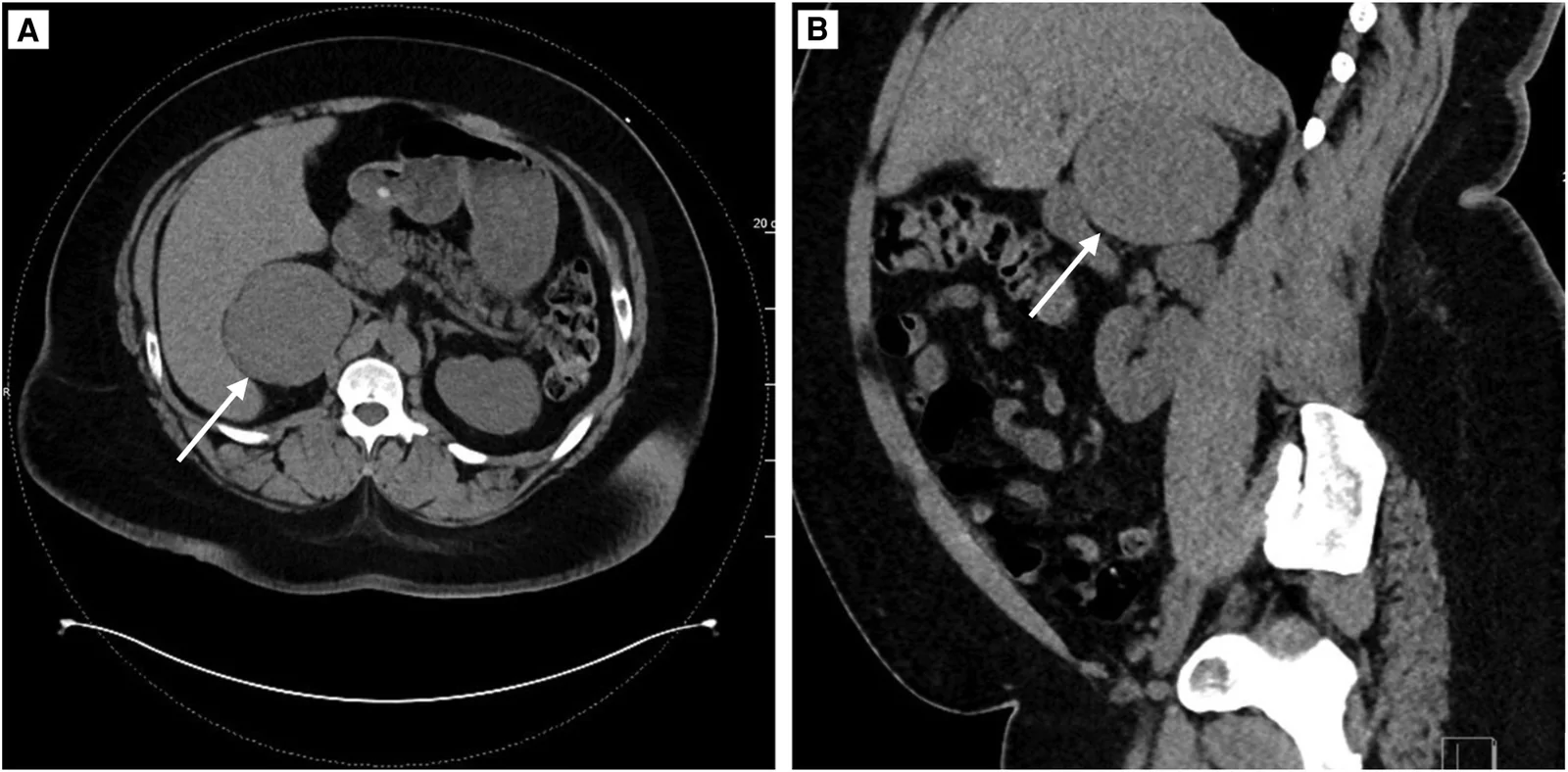
Noncontrast computed tomography of the abdomen showing the right adrenal mass in axial (A) and sagittal (B) planes. The lesion measured 8.4 × 8.3 × 7.9 cm and had enlarged from approximately 3 cm on a chest CT obtained in 2020, approximately 5 years before the current examination. White arrows indicate the adrenal mass.

Because of severe hypertension and rapid glycemic deterioration, a secondary-hypertension evaluation was pursued. Carvedilol had been initiated first during the index hospitalization, before pheochromocytoma was suspected, as part of the initial management of severe hypertension. Terazosin was subsequently added for α_1_-adrenergic blockade when a catecholamine-secreting tumor became a clinical concern. After stabilization in the intensive care unit and resolution of the HHS, plasma metanephrines were obtained on the medical ward. At the time of sampling, the patient was not receiving a continuous intravenous antihypertensive infusion; his oral regimen included carvedilol 25 mg twice daily, nifedipine extended-release 90 mg daily, lisinopril 10 mg daily, and terazosin 2 mg twice daily. Plasma free normetanephrine was 5165.7 pg/mL (SI: 28.2 nmol/L) (reference range, 0-210.1 pg/mL [SI: 0-1.15 nmol/L]), and plasma free metanephrine was 2060.6 pg/mL (SI: 10.45 nmol/L) (reference range, 0-88.0 pg/mL [SI: 0-0.45 nmol/L]). Noncontrast abdominal CT demonstrated an 8.4 × 8.3 × 7.9-cm right adrenal mass with attenuation of 36 Hounsfield units (Fig. 1). There was no local invasion, lymphadenopathy, or radiographic evidence of metastatic disease. A chest CT scan obtained approximately 5 years earlier during evaluation of cough, sore throat, and sinus congestion had incidentally demonstrated a right adrenal lesion measuring approximately 3 cm, indicating substantial interval enlargement.

## Treatment

The patient was treated in the intensive care unit with intravenous fluids and an insulin infusion for approximately 32 hours before transition to basal-bolus insulin. Preoperative optimization required approximately 6 weeks because of difficult blood pressure and glucose control. Initial antihypertensive therapy included hydrochlorothiazide, carvedilol, lisinopril, nifedipine, and terazosin 2 mg twice daily. Doxazosin 2 mg twice daily was subsequently added and titrated to 4 mg twice daily while terazosin was continued. Before surgery, he required 4 to 5 antihypertensive agents and approximately 60 units/day of insulin.

Robotic right adrenalectomy was performed. The mass appeared approximately 12 cm in situ with surrounding tissue, and dissection was technically difficult because of tumor size and body habitus. Severe hypertension occurred during tumor manipulation, followed by profound hypotension after adrenal vein ligation. Estimated blood loss was 1000 mL, and the tumor was removed intact without capsule rupture or spillage. Postoperatively, hypotension required epinephrine, norepinephrine, phenylephrine, vasopressin, mechanical ventilation, and intravenous hydrocortisone.

## Outcome and follow-up

The patient was extubated on postoperative day 1, remained in the intensive care unit for 48 hours, and was discharged after 7 days. At endocrinology follow-up 2 to 3 weeks later, blood pressure was 110/70 mmHg without antihypertensive therapy, and home glucose values were consistently below 130 mg/dL (SI: 7.2 mmol/L) without insulin or other glucose-lowering medications. Postoperative plasma metanephrines were not available. Annual lifelong biochemical surveillance was planned.

**Figure 2:**
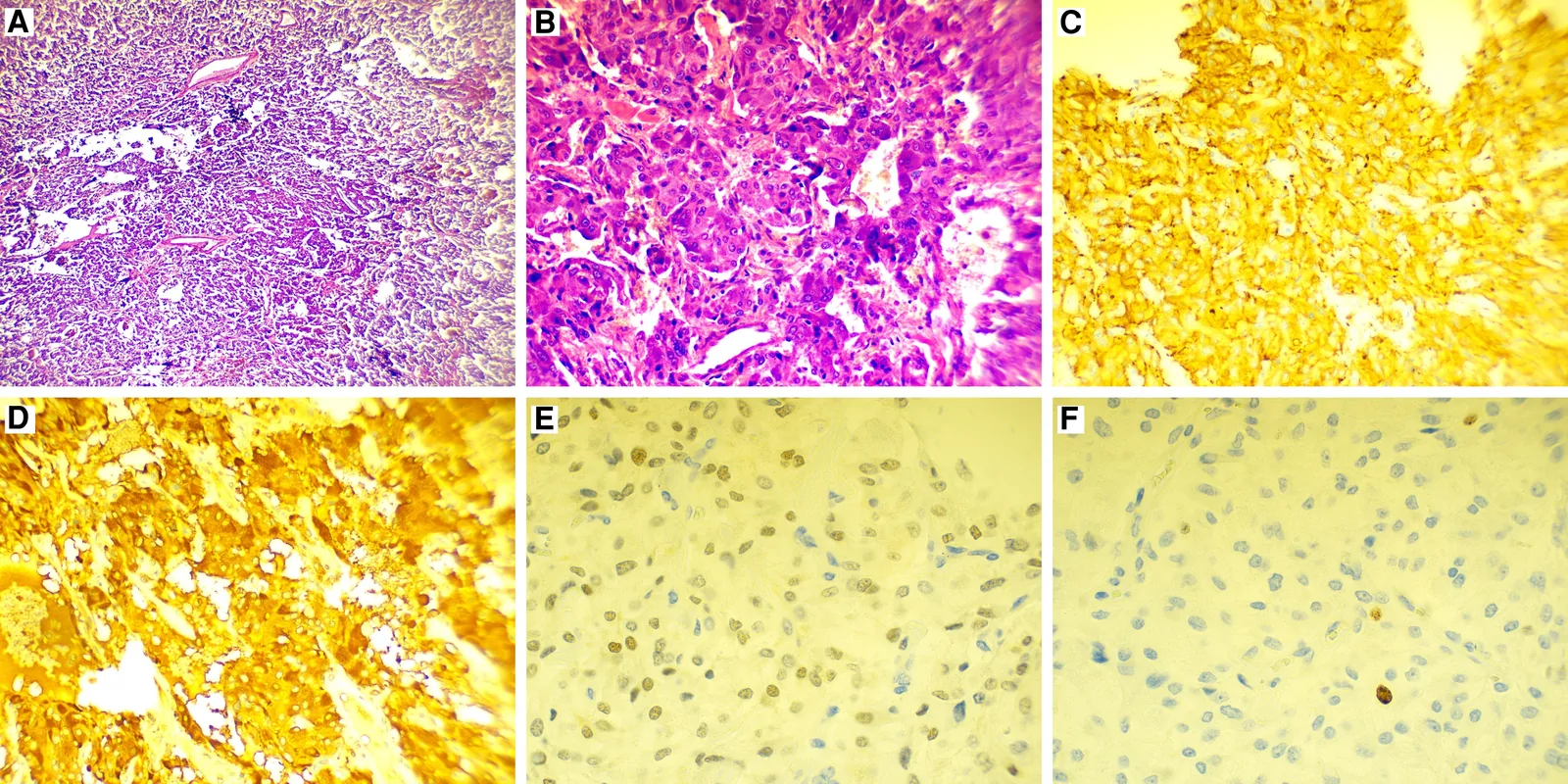
Histopathologic and immunohistochemical features of the right adrenal pheochromocytoma. (A) Hematoxylin and eosin section demonstrating classic nested (zellballen) architecture and a rich vascular stroma (original magnification, ×20). (B) Hematoxylin and eosin section showing predominantly uniform polygonal cells with finely granular cytoplasm, prominent nucleoli, and rare nuclear pseudoinclusions (original magnification, ×200). (C) Diffuse cytoplasmic synaptophysin immunoreactivity (original magnification, ×200). (D) Diffuse cytoplasmic chromogranin immunoreactivity (original magnification, ×200). (E) Nuclear GATA3 immunoreactivity (original magnification, ×400). (F) Ki-67 immunostaining demonstrating a low nuclear proliferative index of approximately 1% (original magnification, ×400).

The adrenalectomy specimen weighed 317 g and contained a well-circumscribed hemorrhagic tumor measuring 9.0 × 9.0 × 6.5 cm. Histologic examination showed classic nested (zellballen) architecture composed predominantly of uniform polygonal cells with prominent nucleoli, rare nuclear pseudoinclusions, finely granular cytoplasm, and a rich vascular stroma (Fig. 2A and 2B). Tumor necrosis, angioinvasion, lymphatic invasion, capsular invasion, and periadrenal soft-tissue invasion were not identified. Tumor cells showed diffuse cytoplasmic immunoreactivity for synaptophysin and chromogranin and nuclear immunoreactivity for GATA-binding protein 3 (GATA3); the Ki-67 index was approximately 1% (Fig. 2C-2F). Margins were negative, and the tumor was staged pT2. Tumor-only molecular profiling reported an *NF1* alteration, but the specific variant designation and classification were unavailable. Germline genetic testing, including evaluation of the succinate dehydrogenase complex iron–sulfur subunit B gene, was ordered as a send-out test but was not completed because the patient did not return for follow-up.

## Discussion

This case links severe hypertension, HHS, and transient focal neurologic dysfunction to catecholamine excess. Hyperosmolar hyperglycemic state is characterized by profound hyperglycemia, hyperosmolality, and dehydration without significant ketoacidosis [6]. In pheochromocytoma, α_2_-adrenergic signaling suppresses pancreatic insulin secretion, whereas β-adrenergic stimulation increases glucagon secretion, thereby promoting hepatic glycogenolysis and gluconeogenesis. Catecholamine excess also contributes to peripheral insulin resistance, in part through reduced glucose transporter type 4–mediated glucose uptake in skeletal muscle [1, 3, 7]. Baseline insulin resistance related to class III obesity likely increased susceptibility. However, the decrease in insulin requirements from approximately 60 units/day to none after adrenalectomy strongly supports catecholamine excess as a major driver of the metabolic decompensation. Published reports of pheochromocytoma presenting as HHS remain sparse [4].

Stroke-like deficits are an uncommon but recognized manifestation of HHS. A systematic review found that negative symptoms, including hemiparesis and aphasia, often improve after correction of hyperosmolarity, supporting a reversible metabolic mechanism [5]. In this patient, normal CT angiography and perfusion imaging and complete symptom resolution with metabolic treatment supported a probable stroke mimic. Because magnetic resonance imaging was not completed, a small acute infarction cannot be excluded with certainty.

The tumor demonstrated classic pheochromocytoma morphology and immunophenotype. Contemporary World Health Organization guidance discourages labeling a primary pheochromocytoma as benign and recommends integrated assessment of stage, invasion, proliferation, molecular findings, and follow-up [2]. Pheochromocytoma of the Adrenal Gland Scaled Score and Grading System for Adrenal Pheochromocytoma and Paraganglioma may provide supplementary prognostic information but have imperfect discrimination and interobserver variability; neither was formally assigned [8]. The reported *NF1* alteration is consistent with the kinase-signaling molecular subgroup of pheochromocytoma and paraganglioma [9], but tumor-only testing without an available variant designation cannot establish pathogenicity or germline status. Incomplete germline evaluation is an important limitation.

Guidelines recommend α-adrenergic blockade and multidisciplinary perioperative planning before resection of a functional tumor [10]. Despite prolonged optimization, this patient experienced hypertension during tumor manipulation and severe hypotension after adrenal vein ligation, illustrating the residual hemodynamic risk associated with large, highly secretory tumors. Metyrosine was not included in the original preoperative regimen because adequate blood pressure control had been achieved with escalating α-adrenergic blockade, and adjunctive inhibition of catecholamine synthesis was not incorporated into the multidisciplinary treatment plan. Nevertheless, metyrosine may be considered in selected patients at high risk for acute catecholamine release, including those with large, highly secretory tumors. In retrospect, given the tumor size, markedly elevated plasma metanephrine concentrations, and intraoperative hemodynamic instability, metyrosine could reasonably have been considered as additional preoperative therapy [11]. The patient's young age and 9-cm tumor support long-term surveillance; annual biochemical testing and lifelong follow-up are appropriate for patients at increased recurrence risk [12]. The absence of postoperative metanephrines and relatively short follow-up are additional limitations. Nevertheless, rapid normalization of blood pressure and glycemia after adrenalectomy provides compelling clinicopathologic correlation.

## Learning points

- Secondary causes of hyperglycemia, including pheochromocytoma, should be considered when abrupt deterioration in glycemic control occurs in the setting of severe or resistant hypertension.
- Hyperosmolar hyperglycemic state may cause reversible focal neurologic deficits that mimic acute ischemic stroke.
- Catecholamine-mediated diabetes and hypertension may resolve rapidly after successful adrenalectomy.
- Preoperative α-adrenergic blockade reduces but does not eliminate the hemodynamic risks of resecting a large functional pheochromocytoma.

## Data Availability

Original clinical data relevant to this case report are included in the published article. No original sequence data were generated or analyzed by the authors. Additional patient-level information is not publicly available because of patient confidentiality.
